# Airborne Coronaviruses: Observations from Veterinary Experience

**DOI:** 10.3390/pathogens10050628

**Published:** 2021-05-19

**Authors:** Paolo Pozzi, Alessio Soggiu, Luigi Bonizzi, Nati Elkin, Alfonso Zecconi

**Affiliations:** 1 Department of Veterinary Sciences, University of Torino, L.go Braccini 2, 10095 Grugliasco (TO), Italy; 2 Department of Biomedical, Surgical and Dental Sciences, University of Milano, Via Pascal 36, 20133 Milano, Italy; alessio.soggiu@unimi.it (A.S.); luigi.bonizzi@unimi.it (L.B.); alfonso.zecconi@unimi.it (A.Z.); 3 Veterinarian, Poultrymed, Oren St. 19, Or Yehuda 6041147, Israel; nati@poultrymed.com

**Keywords:** coronavirus, SARS-COV-2/COVID-19, animals, airborne, diffusion, biosecurity, air filtration

## Abstract

The virus responsible for the pandemic that has affected 152 countries worldwide is a new strain of coronavirus (CoV), which belongs to a family of viruses widespread in many animal species, including birds, and mammals including humans. Indeed, CoVs are known in veterinary medicine affecting several species, and causing respiratory and/or enteric, systemic diseases and reproductive disease in poultry. Animal diseases caused by CoV may be considered from the following different perspectives: livestock and poultry CoVs cause mainly “population disease”; while in companion animals they are a source of mainly “individual/single subject disease”. Therefore, respiratory CoV diseases in high-density, large populations of livestock or poultry may be a suitable example for the current SARS-CoV-2/COVID-19 pandemic. In this review we describe some strategies applied in veterinary medicine to control CoV and discuss if they may help to develop practical and useful strategies to control the SARS-CoV-2/COVID-19 pandemic.

## 1. Introduction

### 1.1. Background

In 2002–2004, a severe acute respiratory syndrome (SARS) outbreak was caused by a coronavirus (CoV) of animal origin (cave-bats) [1]; in 2012, another similar CoV was recognized as responsible for the Middle East respiratory syndrome (MERS-CoV) in the Arab Peninsula [2]. These outbreaks of SARS and MERS surprised medical scientists, but veterinary virologists had previously recognized CoV as a cause of, often fatal, respiratory or enteric disease in animals with interspecies transmission and wildlife reservoirs [3,4,5]. Furthermore, the role of bats as CoV reservoirs and potential spreaders to humans, mammals and birds, was investigated at least since 2005 [6]. There is a consensus on the fact that human coronaviruses (HCoVs), including SARS-CoV and MERS-CoV, are zoonotic pathogens that originated in wild animals. HCoVs are believed to have their origin in bats (excluding the β-CoVs belonging to lineage A, which are thought have their reservoir in rodents) [7].

Coronaviruses are enveloped, single-stranded (ss), positive-sense, non-segmented RNA viruses. With their genome size of 26–32 kilobase (Kb) and a diameter of 60–140 nanometer (nm) (average 125 nm), coronaviruses are considered among the largest RNA viruses. SARS-Cov-2 is ~30 Kb in size, and ~100 nm in diameter [8]. Bovine respiratory CoV is 27–32 Kb in size, and 65–210 nm in diameter, while another enveloped RNA virus of high importance in veterinary medicine, porcine reproductive and respiratory syndrome virus (PRRSV), is only 15.1–15.5 Kb in size, and 45–70 nm in diameter [9,10].

CoVs have been recognized as a source of several diseases in both humans and animals since the ’60s [11], and the importance of these viruses as an important source of epidemic or pandemic diseases was since then [12]. Unfortunately, this feature was confirmed by the emergence of a new strain of coronavirus (SARS-CoV-2/COVID-19), which has been identified as responsible for the current human pandemic affecting more than 150 countries worldwide in the last two years. The α-coronaviruses and β-coronaviruses usually cause respiratory disease in humans and animals, and gastroenteritis in other animals. In addition to the highly pathogenic coronaviruses, such as SARS-CoV-1, SARS-CoV-2, and MERS-CoV, which cause severe respiratory syndrome in humans, four other coronaviruses (HCoV-NL63, HCoV-229E, HCoV-OC43, and HKU1) can infect humans, but in most cases they induce only a mild upper respiratory illness. Based on recent studies, all coronaviruses capable of infecting humans are believed to be zoonotic. SARS-CoV-1, SARS-CoV-2, MERS-CoV, HCoV-NL63, and HCoV-229E appear to have originated in bats; HCoV-OC43 and HKU1 probably originated in rodents [7]. From a genomic perspective, important similarities have also emerged between some epitopes of the spike protein of SARS-CoV-2/COVID-19 with those of dog and bovine [13]. Furthermore, angiotensin-converting enzyme 2 (ACE2), the receptor for SARS-CoV-2 detected in companion, domestic and wild animals, may recognize SARS-CoV-2/COVID-19, suggesting a potential interaction of viral particles with a wide range of ‘host’ cells [14,15].

### 1.2. Coronaviruses in Veterinary Medicine

CoVs are known in veterinary medicine affecting several species, and causing respiratory and/or enteric, systemic disease in mammals and reproductive disease in poultry. Indeed, companion animals, dogs, cats, horses, and ferrets may have CoVs infections, as well as cattle and pigs among livestock, and poultry (chicken, turkey). Other important reservoirs for CoVs are wild or semi-wild animals, and these latter ones are confirmed to be the source for SARS-CoV and MERS-CoV [6], and a probable source for SARS-CoV-2/COVID-19 [16]. The latter was demonstrated as able of reverse spill-over from humans to other animals like dogs and cats, then able of further intraspecies spread as recently demonstrated in farmed fur-minks in several EU countries and in the USA [17].

Table 1 summarizes coronaviruses and animal diseases of usual veterinary interest.

### 1.3. Spread of CoV

Porcine respiratory CoV in swine (PRCV) is known for its aerogenic spread between herds. The virus is shed from nasal secretions for less than 2 weeks, and diffuses through droplets and aerosol; nasal shedding of PRCV in experimentally infected pigs occurs through postexposure until 10 days. PRCV can persist in the herd throughout the year, or it can disappear temporarily in summer and reappear in winter. Waves of infection, without clinical disease, coincide with the rainy season in Europe, and regardless of the population density, PRCV regularly reoccurred in farms in the colder seasons [9]. Population density plays a major role in the spread of PRCV. Indeed, in areas of high swine population density, PRCV can spread several kilometers and the risk of a farm to become infected increases as neighboring herds increase in size within a radius from 2 km–10 km. An epidemiological study conducted on 33 pig farms in Belgium, revealed that the spread of the virus was higher among farms in higher density areas, as follows: 11 out of 11 (100%) farms were PCRV-positive (PCRV +ve) in a high-density area (all the farms were within 4 km^2^), while only 11 out of 22 (50%) of the farms were PCRV +ve in a low-density area (only one or two farms within 12 km^2^) [18]. Similarly, in an epidemiological study conducted in Spain, the frequency of PRCV +ve pigs was positively correlated with herd/population size from 11.66 ± 5.74% +ve pigs (herd with up to 9 sows; 10–100 pigs), to 15.83% ± 6.53% (10–49 sows; 100–550 pigs), to 27.50 ± 7.89% (50–99 sows; 550–1100 pigs), and to 32.50 ± 8.38% (>100 sows; >1100 pigs) [19]. These data underline how CoV spread varies within the population and according to the density of the population itself.

Porcine epidemic diarrhea virus (PEDV), another member of CoVs in pigs, is capable of aerogenic spread; it was detected in aerosols of different sizes and in concentrations ranging from 1.3 × 10^6^ (in particles of 0.4–0.7 μm) to 3.5 × 10^8^ RNA copies/m^3^ (in particles of 9.0–10.0 μm), following experimental infection [20]. In an experimental study, PEDV was detected as airborne for 24 days post-infection, and the virus remained viable and able to re-infect other pigs [20]. Concerning airborne diffusion, higher quantities of PEDV were found associated with larger particles. Indeed, under field conditions, PEDV viral RNA was identified at significantly higher concentrations in particles >3.3 μm in diameter compared with those of 0.4–1.1 μm. The infectious particles with a size <10 μm may increase the risk of serious health implications because they are able to penetrate the lower respiratory tract to establish infection. PEDV showed a statistically higher concentration in large-size particles (>9 μm) vs. particles ranging from 1.1 μm to 5.8–9.0 μm [15]. The aerosols exhaled from human patients with respiratory infections have shown a predominance of different pathogens, both bacteria and viruses, in small-size particles (<5 μm) [21].

Bovine CoV (BCoV), another CoV causing respiratory disease and diarrhea in calves and winter dysentery in adult cattle, considered endemic in cattle populations, is known for its nasal shedding, further than fecal, along 1 to 11–12 days post-infection [22]. Despite direct and indirect contacts (personnel, fomites) being the most likely routes of transmission, the aerosol/airborne transmission route was also confirmed [23,24]. Infected calves were demonstrated shedding BCoV for up to 13 days, with the rapid spread of BCV to all susceptible animals within a unit. The simple separate air-flow equipment, aimed to stabilize air extraction, was not enough to prevent the BCoV spread in separate buildings at a 30 m distance, with the first shedding in nasal secretion demonstrated within 48 h in neighborhood calves groups [23].

Recent outbreaks of SARS-CoV-2/COVID-19 in fur-mink farms confirmed both the airborne transmission of the virus, and its high and quick spread in high-density animal populations, with up to 70–96% serological positivity and up to 30–86.7% virus positive swabs in samples from minks populations of thousands and tens of thousands of individuals [17].

## 2. May Experiences on Livestock CoV Help to Control SARS-CoV-2/COVID-19?

We could look at animal diseases caused by CoVs from the following other perspective: livestock, poultry and fur animals are mainly raised in hundreds and up to tens of thousands of individuals all together. In these conditions, CoVs appear mainly responsible for “population diseases”; while in pets, generally kept as a single animal or in a very small group, CoVs may appear as a source of “individual/single subject disease”, due to their relative isolation from other pets. In fact, canine coronavirus (CCov) again expresses its spread ability to large groups of subjects, inducing epidemics in breeding farms for dogs, or in kennels [25].

Therefore, respiratory CoV diseases in high-density, large populations of livestock or poultry may be a suitable example for the current SARS-CoV-2/COVID-19 pandemic. Livestock and farmed poultry, differently from humans, may be considered “sedentary populations” living for a relatively long time within their farm and within their geographical area. Nevertheless, situations exist in which humans too may be considered a “sedentary population”, for example long-term patients in hospitals and elderly people in residency/nursing homes. In such situations, similar spread mechanisms for respiratory CoVs in livestock and poultry could be considered and may suggest biosecurity measures.

Veterinary medicine also has experience with “high density, non-sedentary populations”, for example when crowding animals in traditional animal fairs, the proximity of the susceptible individuals for a short period with risk of exposure, infection, then spread within the sedentary population when subjects are either back home or purchased to join a new farm/population has been observed. This may resemble some of the following situations in which people gather in a “high density, non-sedentary” way: crowdy discotheques, concert halls, cinemas, crowdy malls, schools, etc. Again, appropriate biosecurity measures should be taken into account also in these cases. In veterinary medicine, the introduction or re-introduction of animals from fairs or outside sources is either avoided or highly monitored and regulated through epidemiological tests, the implementation of particular vaccination plans, and possibly quarantine.

## 3. Biosecurity Measures

Biosecurity implicates a set of management and physical measures designed to reduce the risk of the introduction, establishment and spread of diseases, infections or infestation to, from and within a population. Biosecurity measures aim at preventing the introduction (external biosecurity or bio-exclusion), managing the stability and reducing the spread (internal biosecurity) of disease. Segregation, cleaning and disinfection represent the basic principles of biosecurity in every population (farm, residency, etc.).

Biosecurity plans should identify potential pathways for the introduction and spread of disease in a zone or compartment, and should describe the measures that will be used to mitigate the risk of disease. Veterinary medicine is familiar with biosecurity concepts and the implementation of biosecurity measures. Biosecurity is an integral part of any successful poultry and livestock production; it represents the set of rules and measures taken to prevent the incursion and spread of disease. These have been divided into the following three categories [26]: conceptual, which includes the location for a farm or a population; structural, which covers the physical facilities; and operational, which covers the procedures to be followed by the farm staff and visitors.

### 3.1. Biosecurity in Animal and Human Populations

Relatively to respiratory CoVs, the experience gained in veterinary medicine could be of interest for human medicine as well. Direct contact, airborne transmission, people (personnel, visitors, or suppliers), fomites, and pests (bats), may represent a cluster of biosecurity issues in which veterinary medicine has already developed references. The design of an effective biosecurity plan hinges on an understanding of how disease-causing organisms are introduced and spread, and the identification of any pathogens that might be already present. The best biosecurity plan must be based on understanding the risks to the considered population.

### 3.2. Preventing Introduction (External Biosecurity; Bio-Exclusion)

The airborne spread of CoVs and other RNA viruses was confirmed in both human and animal diseases [13,16,18,19,20]; simple air change/extraction did not prevent the spread of some CoVs [23]. Air filtration or air treatment systems can be an effective way to prevent the aerosol transmission of viruses. The efficacy of filtration is generally measured by the percentage of reduction in dust, droplets and aerosol of different sizes, which may carry viruses and/or bacteria. Table 2 summarizes the filtration classifications of commercially available filtration systems, relative to their efficiency in trapping particles <0.3 µm to 1.0 µm in size.

PRRSV, a tiny RNA single-stranded virus of 15 kb and 50–65 nm, may serve as an example for CoV spread and diffusion within sedentary populations, and among different populations. Studies on the PRRS virus into breeder (boar) units [9] confirmed that air filtration is an effective preventive measure. In a 12-month study in the USA, farms with a history of new PRRSV infections in the previous 7 and 4 years, were equipped with minimum efficiency reporting value (MERV) 14–15 or MERV 13–14 filters, and negative-pressure ventilation systems [28]. These filters are, respectively, 94% and >75% efficient at capturing particles greater than or equal to 0.3 µm in diameter. The filtered herds remained PRRSV PCR-negative, contrary to the non-filtered control herds, which experienced severe clinical episodes of PRRS. According to this experience, the viral concentrations in air resulted higher at low ventilation rates and at higher non-filtered ventilation rates, whereas reducing the air pressure (negative-pressure ventilation) reduced the viral load. The unfiltered farms were at an eight times higher risk of a new PRRSV infection/outbreak than the filtered farms. In a preliminary study [29], a set constituted of a pre-filter, MERV 13–14 filter (90–95% efficiency), MERV 18 filter (high-efficiency particulate air filter (HEPA) with>99.997% efficiency @ 0.3 µm) in sequence, avoided the aerosol transmission of PRRSV from experimentally infected pigs to sentinel pigs in a separate room receiving air flow from infected pigs through the described filtration system. While it may be difficult discussing the logistics of such filtering systems in public buildings from a veterinary point of view, these results could have been helpful in speculating how to implement highly efficient ventilation systems in hospitals with SARS-CoV-2/COVID-19 patients and/or elderly residency/nursing homes, aiming to protect CoV-negative areas with fragile patients or guests that are still SARS-CoV-2/COVID-19-negative.

### 3.3. Managing the Risks of Disease Entering a Population of Elderly Residency/Nursing Homes

While it is again difficult for the authors to enter into the details of SARS-CoV-2/COVID-19 pandemic tolls at private homes, hospitals and nursing homes, it looks less difficult to examine some characteristics of the main populations involved: the elderly residency/nursing home population and long-term hospitalized population. The elderly residency/nursing home population is at high risk. The SARS-CoV-2/COVID-19 pandemic data show that mortality rates are higher within nursing homes (30–39%) which total population in western countries is estimated 2% to 5%. Aerogenic spread of CoVs between animal populations has already been demonstrated [9,18,19,20], but there are also evidences for the current pandemic. Indeed, a neighborhood with a high population density (≥5000 individuals/km^2^) was associated with higher SARS-CoV-2/COVID-19 mortality with respect to a low population density (<150 individuals/km^2^) in elderly people living at their own home [30]. Therefore, the approach based on air filtration or air treatment may represent a very efficient preventive measure, as shown in the previous PRRSV example. The objective should be to prevent the introduction of the virus, in the following ways: outdoor sourced air, positive-pressure, HEPA 17–20 or MERV 16 (no less) (ISOPM1) filtration systems.

Assuming an elderly residency/nursing home population that is SARS-COV-2/COVID-19-free, positive-pressure filtered air would ensure a continuous flow of uncontaminated air, while exhausted air may be channeled, filtered and dispersed. Despite this evidence, only in November 2020 the European Center for Disease Prevention and Control (ECDC) provided a first update to the guidance on heating, ventilation, air-conditioning systems, and air exchanges per hour in closed spaces in the context of the SARS-COV-2/COVID-19 pandemic for confined indoor places. These recommendations fall under the internal biosecurity of indoor environments. The ECDC document, in its Annex 1, summarizes some “National guidelines for heating, ventilation and air-conditioning (HVAC) systems in EU/EEA countries and the UK in the context of SARS-COV-2/COVID-19, complemented by guidelines from other countries and from international professional associations”. These guidelines showed large variability in the recommended measures and suggest poor consistency among the preventive measures suggested. Indeed, natural ventilation increase and/or outdoor fresh air are suggested in nine countries; the use of HEPA filters in four countries; the use of MERV13 (ISOPM1) filters, of ultraviolet germicidal irradiation (UVGI), of minimal air change per hour (ACH) in two countries [31].

### 3.4. Bio-Surveillance; Bio-Containment

SARS-CoV-2/COVID-19 tests should be carried out on elderly residency/nursing home populations. Positive individuals, regardless of their health status, should be immediately removed from shared areas, isolated or hospitalized. In parallel with this strategy, strict internal biosecurity measures for personnel and visitors are highly necessary.

Biocontainment means managing the risks of the disease spreading within a population; many hospitals were equipped with intensive care units (ICU) for SARS-COV-2/COVID-19 patients. These hospitals presented at least two sub-populations, as follow: SARS-COV-2/COVID-19-positive, and “other patients”. The objective should be avoiding the spread of the virus from SARS-CoV2/COVID-19 areas. This result may be obtained by a negative-pressure pre-filtered air system in place at ICUs, then a HEPA 17–20 or MERV 16 filtration system for exhausted air, with no-recirculation. In veterinary models, low/negative environment static pressure reduced the virus concentration [28].

In other patients, the SARS-CoV-2/COVID-19-free areas, positive-pressure HEPA or highest MERV system air filtration should be applied. Moreover, exhausted air must be channeled, filtered and dispersed outdoors. No communication between the two airflow systems should be allowed. In veterinary models, positive pressure to the filtration system reduces the chance of leaks, and the entry of unfiltered air into the facility during the entry and exit of people [29]. Again, the above strict internal biosecurity measures for personnel and visitors are highly necessary.

Recent reports of SARS-CoV-2/COVID-19 positivity in some farmed animals, with reverse spill-over of the virus, would also suggest a bio-surveillance plan aimed at systematically testing workers who are involved in their farming, and hypothesizing common guidelines for epidemiological surveillance in wild, captive, companion animals in general [32].

### 3.5. Personal Protective Equipment (PPE)

These equipment have their parallel in preventive PPE approaches in livestock infections, and they are of high importance in reducing the spread of infections [33]. Masks and hand sanitization, together with social distancing, constituted for around one year the main protective measures suggested and implemented at the population level. Lockdown, curfews have been used in several countries, with different schedules and durations. The efficacy and impact of these approaches on global population health should be carefully assessed to be prepared in the case of a new pandemic [34,35,36].

Other recommendations, such as distancing (1–2 meters according to most countries); “sneezes protection”; “towels and scarfs”; up to “masks” and other generical indications, have a questionable rational and their usefulness is also arguable. In most of the cases, people used “face masks” or “surgical masks” supplied by pharmacies and/or general stores, or self-made “community masks”, or even “fashion masks”. Masks are intended to protect the wearer from splashes and large droplets, and minimize the particles expelled by the wearer himself; in such a perspective, they should be at least surgical type 2 masks. Generic or face masks do not provide adequate protection against airborne contaminants. Respiratory protection is required to prevent transmission via the airborne route. Seals tighter than those of surgical masks are needed around the nose and mouth to prevent inhalation of some airborne particles.

Unlike surgical masks, N95/FPP2 respirators provide a proper tight seal to the face when worn (fit-tested and seal-checked). The N95/FPP2 respirators filter the small particles inhaled by the wearer, therefore they are indicated for sanitary personnel and in veterinary medicine by livestock operators and veterinarians. The N95 respirators are considered to adequately protect against most normal exposures in veterinary practice [37]. Relative to the COVID-19 pandemic, N95/FFP2 should be without a valve, because the valve does not prevent adequate filtration of exhaled breath. Table 3 summarizes EU, USA standards and filtration characteristics of face masks intended for PPE.

**Table 3:** Mask types; EU or USA standards; filtration efficacy requirements.

Mask Type	Standards	Expected Filtration Efficacy—Particles Size	
Self-made masks	none	40–90% aerosols penetrate [38]	
Face masks	none	≥95%—0.3 µm	
Surgical masks	EN ^1^ 14683	Type I: ≥95%—0.3 µm	
		Type II: ≥98%—0.3 µm	
		Type IIR: ≥98%—0.3 µm	
	ASTM ^2^ F2100	Type 1:	≥95%—0.3 µm
			≥95%—0.1 µm
		Type 2:	≥98%—0.3 µm
			≥98%—0.1 µm
		Type 3:	≥98%—0.3 µm
			≥98%—0.1 µm
Respiratory masks	EN ^3^ 149: 2001	FFP1: ≥80%—0.3 µm	
		FFP2: ≥94%—0.3 µm	
		FFP3: ≥99%—0.3 µm	
	NIOSH ^4^ 42–CFR 83	N95-KN95: ≥95%—0.3 µm	
		N99–KN99: ≥99%—0.3 µm	
		N100–KN100: ≥99.97%—0.3 µm	

^1^ EN14683:2019, construction, design, performance requirements and test methods for medical face masks; EU; ^2^ ASTM F2100, standard specification for performance of materials used in medical face masks; USA; ^3^ EN 149:2001, respiratory protective devices—Filtering half masks to protect against particles—requirements, testing, marking; EU; ^4^ NIOSH 42–CFR 83, the National Institute for Occupational Safety and Health, USA.

Were the above indicated recommendations to the population enough? Probably not. The respiratory protection provided by the surgical masks against airborne contaminants was already found to be significantly lower than that provided by the FFPs (or American N) in a UK report from 2008 and in the prevision of a possible influenza pandemic [39]. According to the Centers for Disease Control and Prevention (CDC) of the U.S. Department of Health & Human Services, surgical masks are less effective in filtering small particles and protecting the wearer than N95 respirators. The limited protection to health care professionals (HCP) is due to facial seal leakage. Due to the facial leakage, surgical masks provided only some block rate, as follows: 63% for tiny virus-sized particles when compared to >97% of 0.01 μm particle filtration provided by N95 (26). Especially at the beginning of the pandemic, sanitary personnel in ICUs were wearing multiple masks, and this approach was also suggested for common people; wearing multiple masks may help reduce small-particle penetration, but not as well as an N95 [40]. Indeed, the N95 mask showed a 95% particle reduction (0.02 μm–1 μm) against an 82% reduction observed when wearing five overlapping surgical masks, 78% with three masks, and 74% with two masks. Relatively to N95, it should be underlined that in the USA and in some EU countries, these respirators were indicated for sanitary personnel only, and not for the wider population. Only recently (January 2021), Austria and Germany are making FFP2 masks for the population when in shops and on public transportation mandatory. A “Position paper” from the German Respiratory Society includes, among the rest, FFP2 and FFP3 masks in hospitals’ personal equipment to which personnel should adhere, in order not to increase the risk of infection of the staff [41].

### 3.6. Controversial Measures: Confinements; Lockdowns; Curfews

In livestock and poultry, kept in large numbers, vaccination plans and/or preventing the introduction of pathogens are often associated with the strict confinement or segregation of herd populations. In most of the cases, livestock and poultry may leave the farm only when directed to slaughterhouses, while fur animals are slaughtered on the spot. The introduction of new subjects may be subject to epidemiological tests and/or potential disease-carrier status of newly introduced animals.

In the case of epidemics, a total strict confinement can be imposed on all the susceptible population, with a no-entry and no-leave policy, even for a relatively long time. On purpose, we do not discuss the stamping-out option due to the context.

A further surveillance zone may be imposed surrounding the epidemic area, with again strict control on the movements of animals. The epidemiological pattern of the involved pathogens drives the restrictive measures but, in general, there are no major differences in the measures adopted by different countries in relation to major pathogens of veterinary relevance. How many of these measures can be applied to human populations? Epidemiological implications and economic consequences represent sufficient motivation in veterinary medicine, while “social” implications do not represent a concern as it may happen when dealing with a human epidemic. The fact is that during this current SARS-CoV-2/COVID-19 pandemic, totally different preventive and protective measures, and/or restrictions were suggested or imposed to the people in different countries. Some countries imposed total, repeated lockdowns and night curfews, which were quite hard on the populations involved; in other countries this was not the case at all. A common and shared understanding of the epidemiological characteristics of SARS-CoV-2/COVID-19 should have suggested the implementation of the same control measures, including movement restrictions, if necessary, regardless of borders issues.

How efficacious can these measures can be? Past and recent experiences in veterinary medicine relative to respiratory CoVs [42] indicate that the virus is able to escape biosecurity and control systems, also due to its airborne diffusion; the high-density populations in close environments [15,16] facilitate its spread. High-density populations in relatively open but small spaces may also facilitate CoVs spread, as was the case in intensive fur-minks farms or in cattle farms. According to a systematic review on the transmission of SARS-CoV-2/COVID-19, by the University of California, the chances of getting SARS-CoV-2/COVID-19 in outdoor settings is 18.7 times less than indoor. In outdoor environments, the intensity of contacts, lack of PPE and occasional gathering in close environments were found to be associated with reports of infection [43]. In such a perspective, specific biosecurity measures finalized to the containment of spread events in closed/indoor environments, and the management of crowded populations when outdoor, are probably more appropriate than generalized lockdowns.

### 3.7. Herd/Population Immunity

In veterinary medicine, disease control is based on both trying to prevent the exposure of animals to pathogens and/or, when possible, inducing an immune response in susceptible populations through vaccination. In companion animals, the implementation of vaccination plans and the relative isolation within domestic walls—especially in cats—look effective to the purpose.

Veterinary medicine has good experience with the population vaccination concept and herd immunity. In a veterinary approach, the whole susceptible population is generally vaccinated; vaccination schedules may change according to age, passive immunity interference or productive phase (i.e., pregnancy). “Population or herd immunity” represents the result of the susceptible population vaccination; for example, rinderpest (now eradicated) required a 70%–90% population/herd immunity, while foot and mouth disease requires an 80%. It means that following the vaccination of 100% of susceptible subjects, we expect that an immune response will develop in a significant part of the population (70%–90%), resulting in the protection of the entire population, generally from the disease rather than the infection. The significant reduction in “susceptible/available guests” in which the pathogen can multiplicate and diffuse, will cause it to vanish.

Relative to the CoVs of veterinary interest, only few vaccines are available, as follow: against canine enteric CoV; feline infective peritonitis; neonatal calf diarrhea; transmissible gastroenteritis in pigs; and avian infectious bronchitis.

Puppies and kittens are vaccinated on a regular basis in almost all western countries, but it is a matter of fact that living as pets, relatively isolated from other conspecifics, makes the burden of strict biosecurity easier, unless in epidemic episodes in kennels.

Strict biosecurity measures and scheduled vaccinations of the whole pregnant heifer/cow or gilt/sow populations are implemented respectively to control and reduce the damages induced by neonatal calf diarrhea and porcine transmissible gastroenteritis CoVs, even if these are not considered as airborne.

Infectious bronchitis virus (IBV) may represent an example of difficulties and constraints related to airborne CoVs. The control and reduction in the damages induced by IBV request strict biosecurity protocols and intense vaccination schedules [44], which include the whole population, both egg-layers and/or broilers. Mass vaccinations are executed on the 1st day of age in broilers, or even “in-ovo” during incubation; hens request multiple vaccinations in their life [45]. The emergence of new serotypes and variants continually occurs [45], challenging poultry productions. IBV teaches us about the difficulties in controlling a disease induced by an airborne CoV and its attitude to generate variants. Again, the available strategies for its control remain strict biosecurity, mass vaccination, and the development of new vaccines.

Vaccination campaigns against SARS-CoV-2/COVID-19 in the EU and most western countries started in the last days of December 2020. Countries are proceeding with vaccination at different speeds, according to vaccine supplies and different implementation policies. In Italy, 10% of around 3,000,000 elderly people (>65 years) with “limited functions” are residents of elderly/nursing homes. The ratio between residents and personnel (nurses, assistants, doctors) is estimated to be 4/1–5/1, excluding volunteers and visitors that cannot be estimated. Concerning vaccination, assistants, volunteers, and visitors should all be considered at the same level of the elderly residency/nursing home population; therefore, all of them must be vaccinated.

When vaccination cannot be implemented due to an illness situation or risk of being impaired by the immunological status in some subjects, it should be postponed until the first suitable moment and, in such a situation, fragile individuals should possibly be isolated. In veterinary medicine, short-term postponing of vaccination in sick animals (often isolated in a “sick-bay”), or in pregnant livestock very close to calving/farrowing, is routinely implemented. These subjects will be included in the vaccination program as soon as the situation allows, unless they are removed from the farm. When implementing vaccination plans in animal populations, the objective is that no one is left behind.

While in veterinary population medicine it sounds simply unthinkable to keep non vaccinated subjects (whatever the species) in contact with the population susceptible to disease, the SARS-CoV-2/COVID-19 pandemic revealed an ideologic objection to the vaccination, called the “no-vax” individuals. This group of people introduce a new and hardly manageable variable in the epidemiological scenario. A 5/1–4/1 ratio in residents/personnel in elderly residency/nursing homes, with 20% or 40% “no vax” personnel, will lead to the presence of 3, 3–6, 6 unvaccinated/susceptible subjects out of 100. The impact of people, or HCP, refusing vaccination on the spread of the disease and on the time needed to have a population immunity is unforeseeable. Only recently a no-vax nurse, asymptomatic SARS-CoV-2/COVID-19-positive, was recognized as responsible for a 17 infected persons cluster, of whom 3 died, in an Italian hospital [46].

The experiences concerning IBV vaccination resemble what is observed in human populations as new SARS-COVID-19 variants, from both the clinical and immunization side [47,48,49,50]. Moreover, the recent problems concerning the AstraZeneca (Vaxzevria) vaccine decrease the trust of the population on vaccination strategy as the main tool to control the diseases. All together these conditions make the vaccination program more critical to apply and to manage in humans than in veterinary medicine.

## 4. Conclusions

Coronaviruses have been known for a long time in veterinary medicine, in different animal species. The airborne spread ability of coronaviruses and RNA viruses in general is also already known. In livestock production, prevention or mitigation measures against virus spread within susceptible populations are already in place, including alternative systems of air-flow management and filtration. Biosecurity in veterinary medicine is an integral part of any successful production, representing the set of rules and measures taken to prevent the incursion and spread of disease; some of these rules and measures may be taken into consideration in the current human pandemic. Susceptible populations, especially sedentary populations, should be protected against viral spread through ventilation/air-flow control and the blocking of spreaders. The interpersonal spread of the virus should be abated or mitigated through the use of recognized appropriate and effective PPE, without leaving to improvisation and self-made solutions. Population vaccination and population/herd immunity are also well-known concepts in veterinary medicine, including specific experiences against coronaviruses and/or RNA/airborne spread viruses. In the current pandemic, vaccination of the entire susceptible population is also an important measure to reduce the potential spread of the virus within a susceptible/fragile population.

## Figures and Tables

**Table 1:** Coronaviruses and animal diseases of veterinary interest (from [16], modified). The four genera apparently have a common ancestor dating 10,000 years back (from [7], modified).

Order: Nidovirales, Family: *Coronaviridea* (RNA Viruses; Enveloped; Single Stranded; Positive Sense), Sub-Families: *Ortho Coronavirinea*					
Animal Population	Animal Species	Genera			
		Alphacoronavirus	Betacoronavirus	Gammacoronavirus	Deltacoronavirus
Livestock	*pig*	Transmissible Gastro Enteritis; Porcine Respiratory CoV; Porcine Epidemic Diarrhea; Severe Acute Diarrhea Syndrome;	Porcine Hemagglutinating Encephalomyelitis;		Porcine delta enteric coronavirus (PDCoV);
	*cattle*		Neonatal calf diarrhea; Bovine Respiratory CoV;		
Companion	*dog*	Canine Enteric CoV;	Canine Respiratory CoV;		
	*cat*	Feline Infective Peritonitis; Feline Enteric CoV;			
	*horse*		Equine CoV;		
Avian	*chicken*			Infectious Bronchitis CoV;	wild; farmed game; enteric/respiratory Delta-CoVs
	*turkey*			Turkey Enteric CoV;	

**Table 2:** Filtration efficacy classifications (from filter classification according to previous EN779:2012; current ISO:16890; ASHRAE 52.2.) From [27], modified.

Classification	MERV ^1^ Classification	EN779 Classification and Efficacy in Trapping Particles 0.3–1.0 µm	ISO:16890 Classification and Efficacy in Trapping ≥ 50% of Particles of Indicated Diameter	Example of Contaminants and Sizes
E3	1–4	G1–G4 < 20%	Coarse: at <10 µm; efficacy < 50%) PM10: at <10 µm; efficacy > 50%	dirt, debris, industrial, fiberglass; pollen, dust mites, carpets’ fibers;
	5–8	G1–G4 < 20%	PM2.5: at <2.5 µm	3.0–10 µm mold spores, cooking dusts;
E2	9–12	M5–M6 < 20%		1.0–3.0 µm lead dust, welding dusts;
E1	13	F7 < 75%	PM1: at <1 µm	0.3–1.0 µm (prefilters to HEPA) bacteria, sneezes, smoke;
E1	14	F7 to F8 75–84%		
E1	15	F8 to F9 85–94%	PM2.5: at <2.5 µm	
E1	16	F9 > 95%		
HEPA ^2^	17	F9 99.97% ^3^		particulate matter < 0.3 µm; viruses, carbon dusts;
HEPA	18	99.997%		
HEPA	19	99.9997%	PM1: at <1 µm	
HEPA	20	99.99997%		

^1^ MERV: minimum efficiency reporting value; according to ASHRAE 52.2. ^2^ HEPA: high-efficiency particulate air filter; according to European standard EN 1822-1:2009. ^3^ If 10,000 particles of 0.3 µm are blown into a HEPA air filter, only 3 particles pass through.

## Data Availability

Not applicable.
